# Fatal outcomes following onasemnogene abeparvovec in advanced-stage spinal muscular atrophy

**DOI:** 10.1038/s41434-025-00535-8

**Published:** 2025-04-23

**Authors:** Peerada Pongsakornkullachart, Pimchanok Kulsirichawaroj, Ratcharin Kongkasuwan, Prakarn Tovichien, Settapong Jitwongwai, Supaluck Kanjanauthai, Nutnicha Preeprem, Sivaporn Limpaninlachat, Nisasri Sermpon, Oranee Sanmaneechai

**Affiliations:** 1 Department of Pediatrics, Faculty of Medicine Siriraj Hospital, Mahidol University, Bangkok, Thailand; 2 Siriraj Center of Research Excellence in Neuromuscular Disease, Faculty of Medicine Siriraj Hospital, Mahidol University, Bangkok, Thailand; 3 Department of Rehabilitation Medicine, Faculty of Medicine Siriraj Hospital, Mahidol University, Bangkok, Thailand; 4 Faculty of Physical Therapy, Mahidol University, Nakhon Pathom, Thailand

**Keywords:** Neurological disorders, Diseases

## Abstract

Supported by encouraging trial outcomes, onasemnogene abeparvovec (OA) was authorized for spinal muscular atrophy (SMA). Nevertheless, efficacy of OA in advanced SMA patients remains underexplored. This investigation assessed clinical effectiveness and adverse effects of OA in a cohort including advanced SMA, and compared to historical survival data for SMA type 1 patients in Thailand. We conducted observational cohort study at Siriraj Hospital, Thailand, from May 2019 to April 2022. The study enrolled eight SMA patients receiving OA therapy. The cohort comprised five SMA type 1 patients treated at 16.7 months (6.5–24.9 months) and three SMA type 2 patients treated at 20.3 months (19–31.5 months). Before receiving OA, all Type 1 patients required 24-hour invasive ventilation and feeding support. Post-treatment, Three of five showed gradual improvement in motor scores, but none achieved new motor milestones. Survival rate was not improved, with all experiencing fatalities. Conversely, Type 2 patients exhibited motor score improvement without serious adverse events. OA did not significantly improve clinical outcomes or survival rates in advanced Type 1 SMA. These findings highlight need for additional caution when administering OA to severe SMA Type 1 and more specific guidelines in selecting subgroups for treatment.

## Introduction

Spinal muscular atrophy (SMA) is an autosomal recessive motor neuron disorder marked by hypotonia, progressive muscle weakening, and areflexia. This condition stems from a homozygous deletion of the survival motor neuron 1 (*SMN1*) gene, leading to diminished levels of functional survival motor neuron (SMN) protein [1].

SMA is categorized based on the age of onset and achieved motor milestones. Type I SMA, which constitutes 60–70% of cases, typically appears within the first 6 months of life, and the severity of muscle weakness increases over time. In the absence of intervention, patients with type 1 SMA generally need permanent ventilation by 11 months of age and often succumb to the disease before their second birthday [1, 2]. Type 2 SMA, which is moderately severe, emerges between 6 and 18 months of age and is characterized by hypotonia and proximal muscle weakness, rendering patients unable to walk independently. Additionally, individuals with type 2 SMA frequently develop complications such as kyphoscoliosis and respiratory insufficiency, requiring positive pressure support [1].

Since 2016, the advent of targeted disease-modifying therapies has notably altered the trajectory of SMA. These treatments include nusinersen (Spinraza), risdiplam (Everydi), branaplam (LMI070), and a gene replacement therapy (onasemnogene abeparvovec, Zolgensma), each contributing to significant clinical improvements [3–9].

Onasemnogene abeparvovec (OA) is a single-dose intravenous gene therapy engineered to introduce a functional copy of the human *SMN* gene via the adeno-associated virus serotype 9 (AAV9) vector [9]. OA is indicated for the treatment of SMA patients under 2 years of age with biallelic mutations in the *SMN1* gene [10] or body weight less than 21 kg, independent age [11]. The efficacy of this therapy was underscored by results from phase 3 clinical trials, such as the STR1VE-US study. That study demonstrated a significant improvement in clinical outcomes: 91% of enrolled SMA type 1 patients survived without permanent ventilation at 14 months of age, compared to only 26% in the untreated cohort from the Pediatric Neuromuscular Clinical Research Network [2, 12, 13]. However, due to a scarcity of supporting data, the use of OA is currently limited in advanced SMA patients who are already permanently ventilated [9]. Most clinical and observational studies have concentrated on SMA patients under 2 years of age who maintain adequate respiratory and bulbar functions, whether they are treatment-naive or have previously received nusinersen [12–18].

In Thailand, SMA ranks as the second most common neuromuscular disorder among children [19, 20]. Despite its prevalence, diagnosing SMA in Thailand poses significant challenges. This is primarily due to the lack of a national newborn screening program and limited access to genetic testing. Genetic testing is available only in tertiary medical centers and is not covered by the Universal Coverage Scheme (a government health insurance program). These constraints impede the timely diagnosis of SMA across the country. Previously, specific treatments for SMA were neither approved nor available in Thailand. The care of SMA patients has traditionally depended on supportive interventions, including physical therapy, occupational therapy, and respiratory and nutritional support, which aligns with the most recent guidelines [21, 22]. However, the approval of specific SMA treatments in Thailand, including OA and risdiplam, marked a major advancement in the therapeutic options available for SMA patients.

This real-world observational study aimed to evaluate the outcomes and safety of OA, including in advanced SMA patients in Thailand, and to compare the survival status of these patients with that of historical comparators within the Thai context. In this study, OA was provided through the Zolgensma Accelerated Access Program and the Global Managed Access Program to five SMA type 1 patients and three SMA type 2 patients.

## Methods

### Study design and patient population

This observational cohort study included all SMA patients who were treated with OA between May 2019 to April 2022 at Siriraj Hospital, Mahidol University, Thailand. All patients were confirmed to have genetic biallelic mutations in exons 7 and 8 of the *SMN1* gene, were under the age of 2 years at the time of program registration, and had no contraindications for OA [9]. Risks and benefits were thoroughly discussed with the parents, and informed consent was obtained before treatment. No exclusion criteria were applied.

Additionally, our study incorporated a historical comparator group comprising 21 patients who were diagnosed with SMA type 1, had not received any specific treatment, and were under follow-up at Siriraj Hospital from 2006 to 2021. This group was used for comparative analysis. The study protocol was approved by the Siriraj Institutional Review Board (MU-MOU CoA 405/2022), also the historical comparators data was approved by the Siriraj Institutional Review Board (Si CoA 860/2021), and the research was conducted in accordance with the principles of the Declaration of Helsinki.

### Procedure

Baseline laboratory evaluations were conducted within 2 weeks before drug administration. These tests were comprehensive: a complete blood count, liver function tests, cardiac enzymes (troponin-I or troponin-T), blood chemistry, and coagulogram. In scenarios where troponin-T was unavailable, troponin-I was utilized as an alternative for monitoring cardiac enzyme levels. Additionally, pretreatment echocardiography was performed to assess cardiac function.

The patients were admitted to the hospital 1 day before the infusion and were transferred to the pediatric critical care unit on the day of infusion. OA was delivered as a single 60-minute intravenous infusion at a dosage of 1.1 × 10^14^ vector genomes per kilogram (vg/kg) of body weight through a peripheral vein [9]. Following infusion, patients were observed in the critical care unit for 4 h before being moved to a general department, where they remained for 48 h of inpatient monitoring prior to discharge.

All patients received prednisolone starting 24 h before the infusion at a daily dose of 1 mg/kg. It was continued for at least 30 days postinfusion, with dosage adjustments and tapering based on liver function test results. Clinical and laboratory evaluations were also integral to the posttreatment follow-up. During the first three months post-infusion, all patients underwent weekly laboratory tests for the first month, followed by tests every two weeks for at least the subsequent two months. These tests were conducted either at Siriraj Hospital or at designated tertiary care facilities, with laboratory results sent back to Siriraj Hospital for evaluation. Additionally, all adverse events (AEs) were reviewed and managed by a pediatric neurologist and pediatric subspecialty accordingly at Siriraj Hospital. For motor function score assessments and ongoing standard-of-care treatment, all patients were scheduled for follow-up visits at the Neuromuscular Clinic at Siriraj Hospital every three months, where they received standard of care in accordance with the latest SMA guidelines. This standard care included pulmonary function assessment and ventilator titration by a pediatric pulmonologist, nutritional support supervision by a pediatric nutritionist, and rehabilitation through oral and chest physiotherapy.

### Functional and laboratory outcomes

Motor function was systematically assessed prior to treatment initiation and subsequently every 3 months until the final follow-up. For patients under 2 years of age, motor function was measured using the Children’s Hospital of Philadelphia Infant Test of Neuromuscular Disorders (CHOP-INTEND, total score range 0–64) and the Hammersmith Infant Neurological Examination–Module 2 (HINE-2, total score range 0–26). Patients aged 2 years or older or those who achieved the maximum score on the CHOP-INTEND were evaluated using the Hammersmith Functional Motor Scale–Expanded (HFMSE, total score range 0–66), the 32-item Motor Function Measure (MFM32, total score range 0–100), and the Revised Upper Limb Module (RULM, total score range 0–37). For patients aged 18 months to less than 2 years, a combination of the CHOP-INTEND, HFMSE, and MFM-32 scales was used to monitor motor function during this transitional age group. Permanent ventilation was defined as (1) tracheostomy or assisted ventilation for ≥ 16 h per day continuously for more than 3 weeks or (2) continuous intubation for 3 weeks. Survival status was calculated based on the time to death for all SMA Type 1 patients, including both OA treatment groups and historical comparators.

Follow-up laboratory assessments were conducted to monitor adverse drug reactions, including hepatotoxicity, cardiac toxicity, and thrombocytopenia. Hepatotoxicity was classified as mild (≥3 to <5 times the upper limit of normal [ULN]), moderate (≥5 to <20 times ULN), or severe (≥20 times ULN) [23]. Thrombocytopenia was defined as a platelet count less than 75 × 110^3^/L [24]. Cardiac troponin values greater than 0.05 ng/ml were considered elevated [25].

### Statistical analysis

Categorical data are displayed as counts and percentages, while continuous variables were first subjected to a normality test using the Shapiro–Wilk test. Continuous data are presented as the means with standard deviations or medians with ranges, depending on their distribution. Survival analysis was performed using the Kaplan–Meier method, and the data were compared with those of historical comparators. All the statistical analyses were performed with IBM SPSS Statistics (version 26) and Stata 18. A *p* value less than 0.05 was considered to indicate statistical significance.

## Results

### Clinical characteristics

**Fig. 1:**
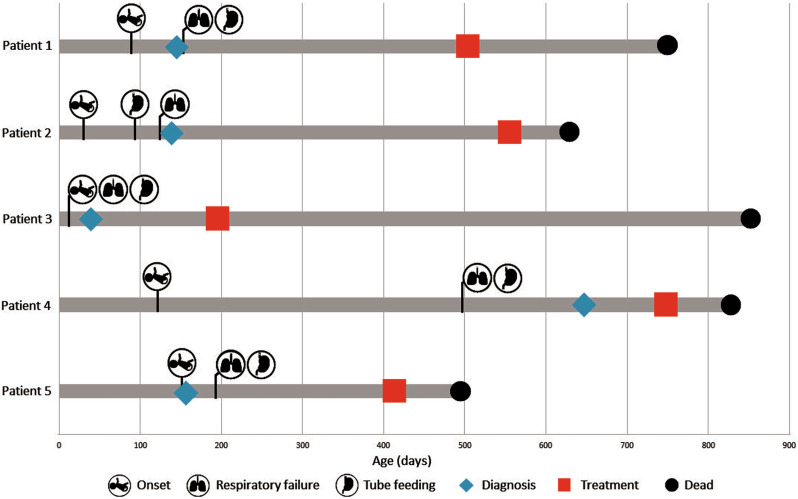
Disease progression summary of SMA type 1 patients treated with onasemnogene abeparvovec.

Eight patients were treated with OA at Siriraj Hospital, Thailand, during the study period. Among these patients, five were diagnosed with SMA type 1, and three were diagnosed with SMA type 2 (Table 1). The median follow-up duration was 220 days. The disease progression among the SMA type 1 patients is detailed in Fig. 1. These patients experienced symptom onset at a median age of 3 months (range 1–5 months). They were diagnosed at approximately 4.8 months (range 1.4–15.3 months) and received treatment at an average age of 16.7 months (range 6.5–24.9 months). All patients with SMA type 1 had two copies of the *SMN2* gene. The median weight at the time of OA administration was 12.1 kg (range 5–14.3 kg). The median CHOP-INTEND score at the baseline visit was 5 (range 1–10). Prior to treatment, all SMA type 1 patients required a tracheostomy for permanent continuous 24-h respiratory support and non-oral feeding support via either gastrostomy or a nasogastric tube. None of the patients had received non-invasive respiratory support prior to treatment due to respiratory failure occurring before the diagnosis was made. The median age at the onset of permanent ventilation and non-oral feeding support was 5.1 months (range 0.4–16.4 months). Patient 3 was diagnosed with protein-energy malnutrition and received nutritional support through a gastrostomy under the supervision of pediatric nutritionist. None of the patients had received nusinersen or risdiplam before treatment with OA. For patients with SMA type 2, symptom onset occurred at a median age of 8 months (range 7–11 months). The patients were diagnosed at a median age of 14.2 months (range 13.7–18.1 months), and treatment was initiated at approximately 20.3 months (range 19–31.5 months). All patients with SMA type 2 had three copies of the *SMN2* gene. Their median weight at the time of dosing was 10.4 kg (range 6.9–16.1 kg), and the median CHOP-INTEND score at baseline was 39 (range 28–54). Patient 8 was diagnosed with protein-energy malnutrition and was under the care of a pediatric nutritionist for improvement of her nutritional status. None of the SMA type 2 patients required respiratory or feeding support before receiving treatment.

**Table 1:** Patient demographics and characteristics treated with onasemnogene abeparvovec for SMA type 1 (Patients 1–5) and type 2 (Patients 6–8).

Patient	Sex	SMA type	SMN2 copy number	Onset of Symptom (month)	Age at Diagnosis (month)	Age at treatment (month)	Weight (kg)	Respiratory Support	Feeding Support	CHOP-INTEND score	Age at last follow-up^a^ (month)	Follow-up time^b^ (month)	Status
1	F	1	2	3	4.8	16.7	12.1	Permanent	NG	4	24.7	8.2	Dead
2	M	1	2	1	4.5	18.5	14.3	Permanent	GT	1	20.6	2.4	Dead
3	M	1	2	1	1.4	6.5	5	Permanent	GT	5	28.1	21.8	Dead
4	F	1	2	4	15.3	24.9	12.3	Permanent	NG	10	27.4	2.9	Dead
5	F	1	2	5	6.9	13.7	8.3	Permanent	GT	6	16.4	3	Dead
6	M	2	3	8	18.1	19	10.4	No	No	54	40.1	21.2	Alive
7	F	2	3	11	14.2	31.5	16.1	No	No	28	49.6	18.1	Alive
8	F	2	3	7	13.7	20.3	6.9	No	No	39	26.6	6.3	Alive

Permanent ventilation: (1) tracheostomy or assisted ventilation for ≥16 h per day continuously for more than 3 weeks or (2) continuous intubation for 3 weeks.

*F* female, *GT* gastrostomy tube, *M* male, *NG* nasogastric tube.

^a^Age at last follow-up: The age at last visit (survivors) or at death (deceased).

^b^Follow-up time: The period from treatment to the last follow-up (survivors) or death (deceased).

Regarding the 21 patients in the historical comparator group, all experienced respiratory failure and death, with 11 of them receiving mechanical ventilation. Some parents opted against invasive procedures, such as intubation, due to the natural course of the disease before treatment was provided in Thailand.

### Motor outcome and survival status

#### SMA

**Fig. 2:**
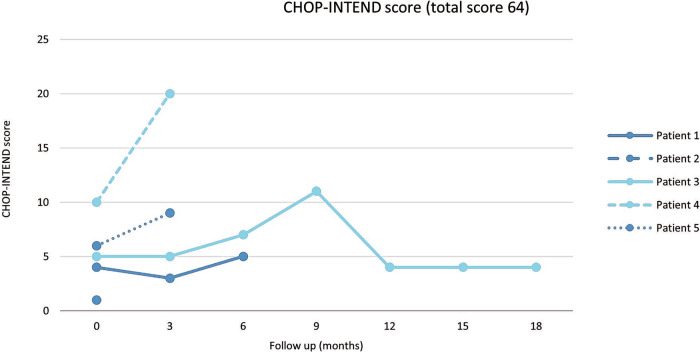
CHOP-INTEND scores of SMA type 1 patients.

Three out of five SMA type 1 patients showed an increase in CHOP-INTEND score (Fig. 2), with two of them achieving a greater than ≥4 point increase, indicating improvements in horizontal movement of the wrist, ankle, knee, and hip. Specifically, Patient No. 4 demonstrated a 10-point increase over a 3-month period. The scores remained stable for the two patients who were followed for more than 6 months after treatment (Patients No.1 and No.3). Tragically, all patients with SMA type 1 died during the follow-up. The median age at death was 24.7 months (range 16.4–28.1 months). Three patients (Patients No. 2, 4, and 5) passed away 2.7 months after treatment with OA and during the tapering of prednisolone. These patients were found to be unresponsive and cyanotic at home and suffered cardiac arrest before reaching the hospital. Despite evidence of transaminitis in their last follow-up laboratory tests, which showed a decreasing trend, cardiac enzyme levels were not elevated. Additionally, none of these patients had any clinical signs suggestive of infection. Another patient (Patient No. 1) died 8.4 months after treatment. The cause of death was suspected to be sepsis secondary to infectious gastroenteritis. Symptoms included mucus, bloody stools, and stupor before the patient passed away at home. The final patient (Patient No. 3) died 21.7 months after treatment from community-acquired pneumonia. No autopsies were conducted on any of the patients due to prevailing cultural beliefs in Thailand.

**Fig. 3:**
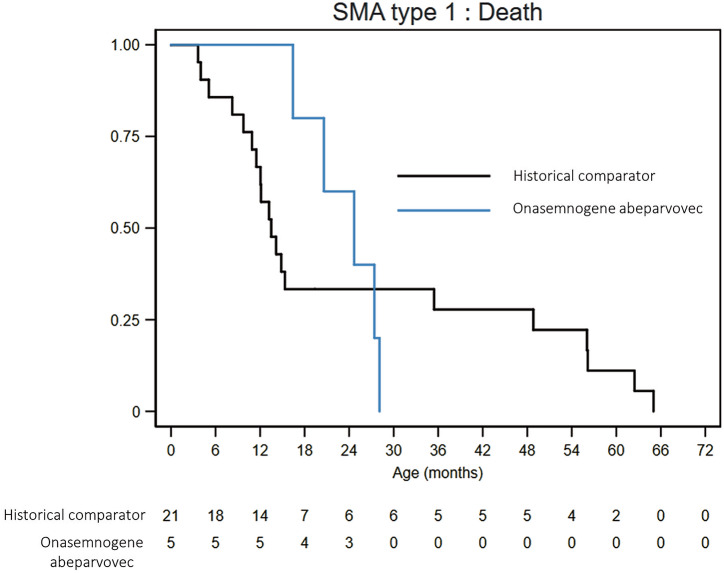
Survival status of SMA type 1 patients.

The survival analysis contrasted the SMA type 1 patients treated with OA to the historical comparators (Fig. 3). The median time to death for the treated group was 24.7 months (range 16.4–28.1 months). This duration did not significantly differ from that of the historical comparators group (*n* = 21, median 13.5 months, range 3.6–65 months, *p* = 0.87).

#### SMA type 2

**Fig. 4:**
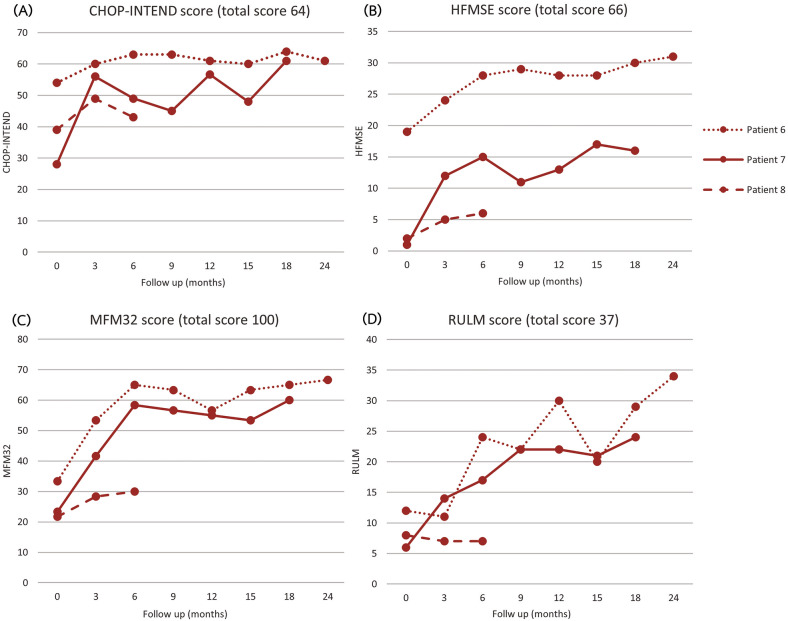
Motor function scores of SMA type 2 patients. **A** CHOP-INTEND score, **B** HFMSE score, **C** MFM32 score, **D** RULM score.

For patients with SMA type 2, there was a gradual increase in motor scores (CHOP-INTEND, HFMSE, MFM32, and RULM) during the follow-up period (Fig. 4). All patients retained the ability to sit independently, and one patient progressed to standing with assistance 3 months posttreatment at the age of 2.6 years. Furthermore, all patients maintained normal oral and swallowing abilities. One patient required initiation of nighttime noninvasive ventilation support due to adenotonsillar hypertrophy, which led to severe obstructive sleep apnea.

### Safety outcomes

All patients experienced adverse events, with serious adverse events occurring in five patients (all SMA type 1). These serious events included the deaths of five patients during the follow-up period. Additionally, three patients (37.5%) developed pneumonia and were treated with intravenous antibiotics. Another two patients (25%) experienced emesis within the first 3 days following treatment. The vomiting was managed without the need for antiemetic medication or intravenous hydration.

The most frequent adverse event observed was transaminitis, which affected every participant. Two distinct peaks of liver enzyme elevation were identified. The initial peak occurred between day 7 and day 14 posttreatment (Aspartate aminotransferase (AST) median 90 U/L, range 50–319 U/L; Alanine aminotransferase (ALT) median 64 U/L, range 23–598 U/L). The second peak occurred at approximately 6 weeks posttreatment (AST median 90 U/L, range 34–301 U/L; ALT median 70 U/L, range 32–445 U/L). No significant increases in Gamma-glutamyltransferase (GGT) or bilirubin were observed. Importantly, no cases of coagulopathy, hepatic encephalopathy, severe hepatitis, or hepatic failure were reported in this cohort.

Cardiac surveillance, including measurements of cardiac enzymes and echocardiograms, was conducted for all participants. Four patients were monitored using troponin-I, while the remaining four were assessed with troponin-T. Baseline cardiac enzyme levels were above the normal limits, with troponin-I levels at 51.4 ng/L (range 2.8–98.7 ng/L) and troponin-T levels at 52.8 ng/L (range 16.7–65.8 ng/L). Clinical evaluations and echocardiograms performed before treatment initiation showed that all the results were within normal ranges. Throughout the three-month follow-up, fluctuations in cardiac enzyme levels were noted, albeit without accompanying clinical symptoms.

Additionally, all patients experienced a transient decrease in platelet counts approximately 7 days after dosing (median nadir platelet count 209 × 10^3^/L, range 129–252 × 10^3^/L). The decrease occurred without clinical bleeding, and the values returned to baseline by week 3. No instances of thrombocytopenia or thrombotic microangiopathy were observed in this study.

### Corticosteroid adjustment

A prednisolone regimen of 1 mg/kg daily was initiated for all patients, with a tapering schedule commencing after week 4, guided by liver enzyme levels. The median duration of prednisolone therapy was 75 days (range 57–146 days). None of the patients required an increase in the prednisolone dosage beyond 1 mg/kg per day or a transition to intravenous methylprednisolone. One patient discontinued prednisolone on day 90 postdosing; however, due to a rise in liver enzyme levels, the administration of the medication was resumed on day 134 and continued until day 190.

## Discussion

This single-center, real-world observational study examined the outcomes of five SMA type 1 patients and three SMA type 2 patients who received OA gene replacement therapy over a three-year period. Our investigation predominantly addressed advanced-stage SMA type 1 patients whose treatment was delayed due to drug authorization and transportation issues. Unfortunately, all patients in this advanced group died, highlighting the critical need to reevaluate the timing and appropriateness of gene therapy in such patients.

Symptom onset in our SMA type 1 patients occurred at 3 months, with diagnosis at approximately 4.8 months. These durations closely mirror those of the Pediatric Neuromuscular Clinical Research Network untreated cohort, which reported symptom onset at 3 months and diagnosis at 6 months [2]. However, there was a notable delay in diagnosis compared to the STR1VE study (symptom onset at 1.8 months and diagnosis at 2.2 months) and other treated cohorts [12–15, 18].

In addition, the age at treatment with OA in our SMA type 1 cohort was 16.7 months, with all patients in the advanced stage and needing permanent respiratory support and non-oral feeding. This marked a substantial delay in the treatment of patients and the severity of their condition at the time of treatment compared to the STR1VE study and other cohorts [12–18].

In contrast, for our patients with SMA type 2, symptoms began at 8 months, with diagnosis at 14.2 months. These values are consistent with those of the Pediatric Neuromuscular Clinical Research Network cohort for SMA type 2 (onset at 9.6 months and diagnosis at 13.2 months) and other treated cohorts [15, 16, 18, 26]. The age at treatment in our SMA type 2 group was 20.3 months, which represented a delay compared to other treated cohorts [15–18].

The delayed diagnosis and treatment of SMA in our cohort are attributable to several factors. Primarily, the absence of a national newborn screening protocol for SMA significantly hampers early detection, which is crucial for prompt intervention. Additionally, genetic testing for SMA in Thailand is largely limited to tertiary medical centers. It is also not included in Thailand’s Universal Coverage Scheme, necessitating referrals for definitive diagnosis and consequently prolonging the process.

The introduction of SMA gene therapy in Thailand has also faced delays. They are chiefly due to the newness of the authorization process and the logistical challenges associated with the importation and distribution of the treatments. Moreover, other specific SMA treatments, such as nusinersen, are unavailable in Thailand, limiting therapeutic options for patients. These obstacles contribute to the progression of the disease to more advanced stages before effective treatment can be administered.

Addressing these challenges involves enhancing early detection, expediting diagnosis, and improving the accessibility of treatments. Establishing a national newborn screening program for SMA, expanding genetic testing capabilities beyond tertiary centers, and integrating these services into the national Universal Coverage Scheme could significantly mitigate these delays and improve outcomes for SMA patients in Thailand.

After receiving gene replacement therapy, our SMA type 1 patients showed modest improvements in CHOP-INTEND scores, particularly in the grade 2 (gravity-eliminated) limb function, as well as in ankle, knee, and hip movement. However, the patients continued to experience profound weakness, and no new motor milestones were attained post-intervention. These findings are consistent with previous reports of treatment in patients with severe forms of SMA [27]. There was no statistically significant difference in survival outcomes compared to the historical comparators. Critically, all SMA type 1 patients suffered severe adverse events, including fatalities. In contrast, the STR1VE study and other treated cohorts reported enhancements in both motor scores and survival rates, accompanied by a considerably lower incidence of serious adverse events [12–18]. The treatment delays in our cohort, leading to advanced disease stages at the time of intervention, resulted in less favorable outcomes. Additionally, our patients required invasive permanent ventilation, on one patient suffered from protein-energy malnutrition. Furthermore, our SMA type 1 patients were not eligible for the STR1VE study, limiting the ability to make direct comparisons. The patient’s status before starting treatment is crucial to magnifying the outcomes. Moreover, the limited observation period, due to patient fatalities, may have hindered the full response to onasemongene abeparvovec. These findings emphasize the vital importance of early treatment initiation in SMA patients and to optimize the patient’s condition prior to treatment to improve treatment effectiveness patient prognoses.

All SMA type 1 patients in our study died during the follow-up period. Among these, one patient’s death was attributed to community-acquired pneumonia, but the specific causes of death for the other four patients remain unclear. Laboratory tests, including cardiac and liver enzyme tests, indicated no significant abnormalities prior to death. Infections and secretion obstructions are considered potential causes of death; however, the lack of autopsy reports precludes definitive conclusions about whether these were related to gene therapy complications or the natural progression of SMA. This situation underscores the need for healthcare systems that reimburse onasemnogene abeparvovec to acknowledge that, in addition to the lack of significant clinical improvement, serious adverse events such as death may occur. This study contributes to the growing body of evidence suggesting that gene therapy should be administered with cautioned to SMA type 1 patients with severe muscle weakness and those who are intubated, highlighting the complex risk-benefit considerations in this group. Several fatalities have also been reported in other studies involving OA. For instance, in the STR1VE study, which included 63 patients, two deaths occurred at 12 and 171 days posttherapy, both of which were due to respiratory failure [12, 13]. In the Global Managed Access Program, which involved 102 patients, three fatalities related to respiratory complications were recorded between 80 and 106 days after treatment, all related to respiratory complications [24, 28]. Importantly, these deaths were determined to be unrelated to the gene therapy itself.

The SMA type 2 patients in our study exhibited more favorable outcomes, mirroring findings from other research [15–18]. Improvements in motor scores were observed over time, and notably, one patient achieved new motor milestones. Additionally, no serious adverse events were reported in these patients, aligning with outcomes documented in similar cohorts.

Posttreatment monitoring in our cohort revealed common adverse events, including transient decreases in platelet counts and instances of transaminitis. The most frequently observed adverse event was mild to moderate anicteric transaminitis, characterized by two peaks of enzyme elevation that did not coincide with any clinical symptoms, consistent with previous studies [12–18]. No specific treatments were required apart from adjustments to the prednisolone dosage. Despite the observed decrease in platelet counts, none of our patients developed thrombocytopenia, a condition that has been reported in other studies with varying frequencies ranging from 9% to 78% [12–18]. This discrepancy highlights the variability in patient responses to gene therapy and underscores the importance of close clinical and laboratory monitoring to effectively manage and mitigate potential adverse effects.

Furthermore, all patients in our study presented with abnormal baseline cardiac enzyme levels. Despite this, no increases in enzyme levels or clinical cardiac abnormalities were observed posttreatment, consistent with data from other treated cohorts [14–18]. Several studies have similarly reported elevated plasma levels of cardiac enzymes, including both troponin-I and troponin-T, among SMA patients without any overt cardiac symptoms. One study reported a median troponin-I level of 39.5 ng/L (range 4–1205 ng/L) and troponin-T levels that were 3–10 times greater in 16 SMA patients (80 ± 39 ng/L, range 43–143 ng/L), with the highest values noted in SMA type 1 patients [29, 30]. Adult studies have indicated increased troponin-T in patients with muscle diseases, suggesting that cardiac troponin-T may be re-expressed during chronic skeletal muscle repair mechanisms. This hypothesis could explain the elevated cardiac enzymes observed in SMA patients despite the absence of cardiac abnormalities [31].

Nonetheless, this study has limitations, particularly the small sample size, which limits the ability to widely generalize the findings. Moreover, no autopsies were performed on the deceased patients, which would have helped to definitively ascertain whether their deaths were related to the treatment or were a natural progression of the disease. This gap in data is due primarily to the cultural practices prevalent in Thailand.

Given the uniformly fatal outcomes observed among the advanced SMA type 1 patients in this study, further research in this specific patient population should be approached with caution at this time. Our findings underscore the need for careful patient selection and management in future clinical applications of gene therapy for SMA.

The future of SMA management in Thailand hinges on early diagnosis, which is achievable through the development and implementation of a comprehensive newborn screening program. Further research into the outcomes of early intervention is crucial, as it represents a significant step toward integrating SMA treatment within Thailand’s Universal Coverage Scheme. This integration would enhance the quality of life for SMA patients and significantly reduce the overall burden of the disease on the nation.

## Conclusion

This study presents real-world outcomes of OA therapy in advanced SMA patients within the context of a developing country characterized by delayed diagnoses and restricted access to alternative treatments. Our findings revealed an improvement in motor scores but no significant progress in motor milestones or survival rates, with all SMA type 1 patients ultimately succumbing to the disease. Therefore, this investigation underscores the imperative of initiating treatment early in the course of SMA and prompts a reevaluation of the use of OA in advanced SMA type 1 patients, particularly permanently ventilated patients. These results highlight the need for cautious and targeted application of gene therapy in this vulnerable patient population, ensuring that treatment approaches are timely and appropriate.

## Supplementary information

supplementary material

## Data Availability

All datasets and data article sited in this manuscript were included in the reference list. Share data is available.
