# Supplementary material for: Fatal outcomes following onasemnogene abeparvovec in advanced-stage spinal muscular atrophy
Source: Gene Ther. 2025 Apr 23;32(5):553–60. doi: 10.1038/s41434-025-00535-8 (PMC12518124; doi:10.1038/s41434-025-00535-8)

**Supplementary figure legends**

**Figure S1** Postdosing enzyme levels. **(A)** Aspartate aminotransferase (AST), **(B)** alanine aminotransferase (ALT). SMA type 1 in blue and type 2 in red.

**Figure S2** Platelet counts following onasemnogene abeparvovec administration. SMA type 1 in blue and SMA type 2 in red.

**Figure S3** Prednisolone dosage adjustments over time

**Figure S1** Postdosing enzyme levels. **(A)** Aspartate aminotransferase (AST), **(B)** alanine aminotransferase (ALT). SMA type 1 in blue and type 2 in red.


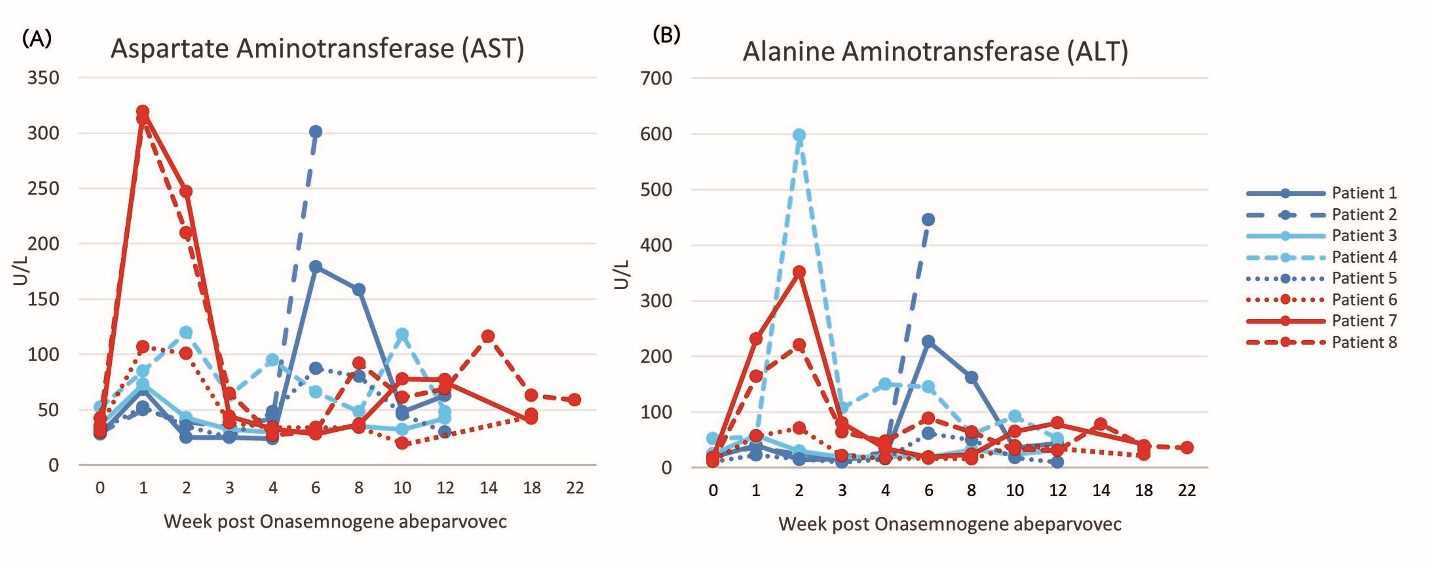


**Figure S2** Platelet counts following onasemnogene abeparvovec administration. SMA type 1 in blue and SMA type 2 in red.


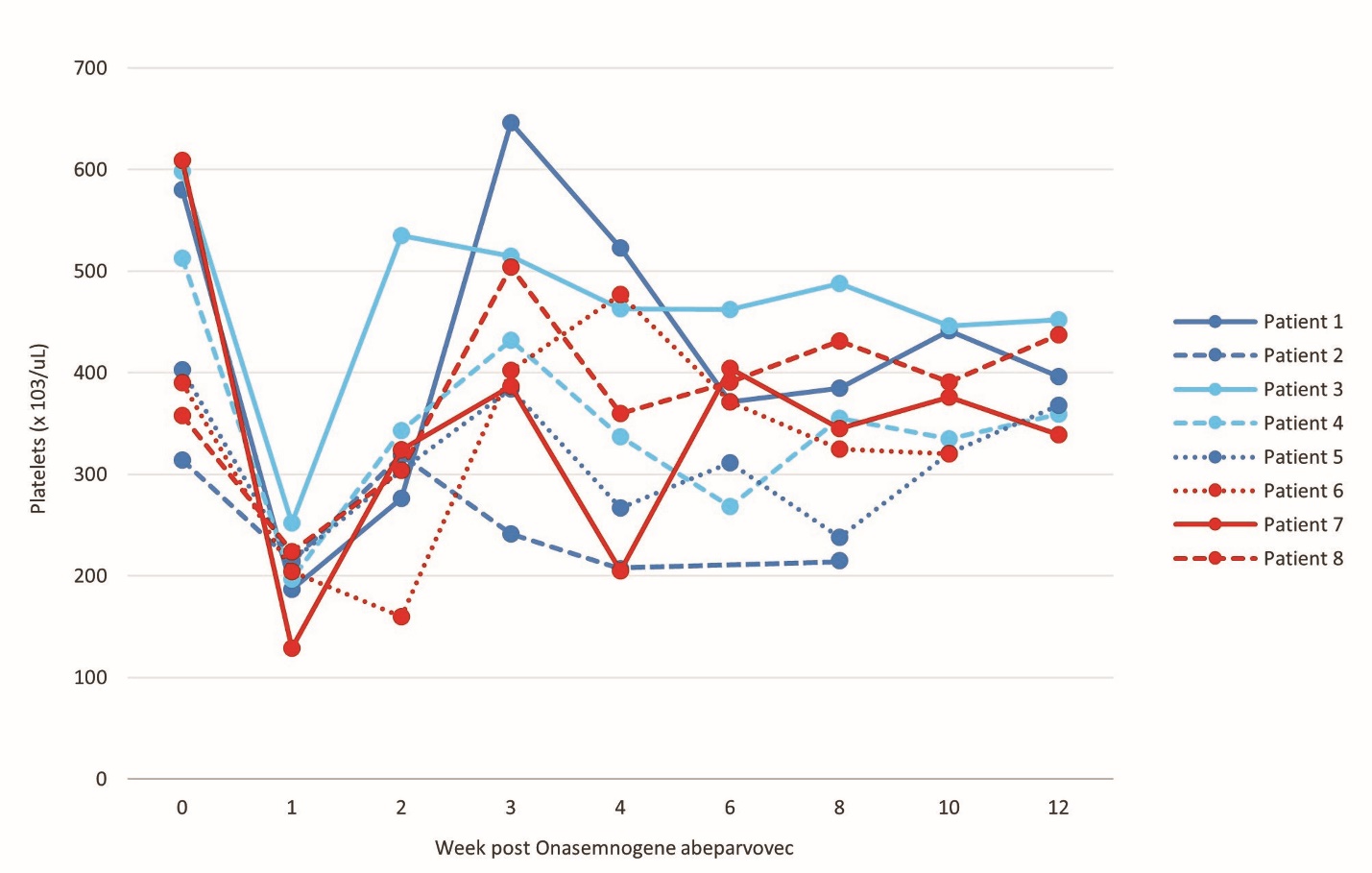


**Figure S3.** Prednisolone dose adjustment


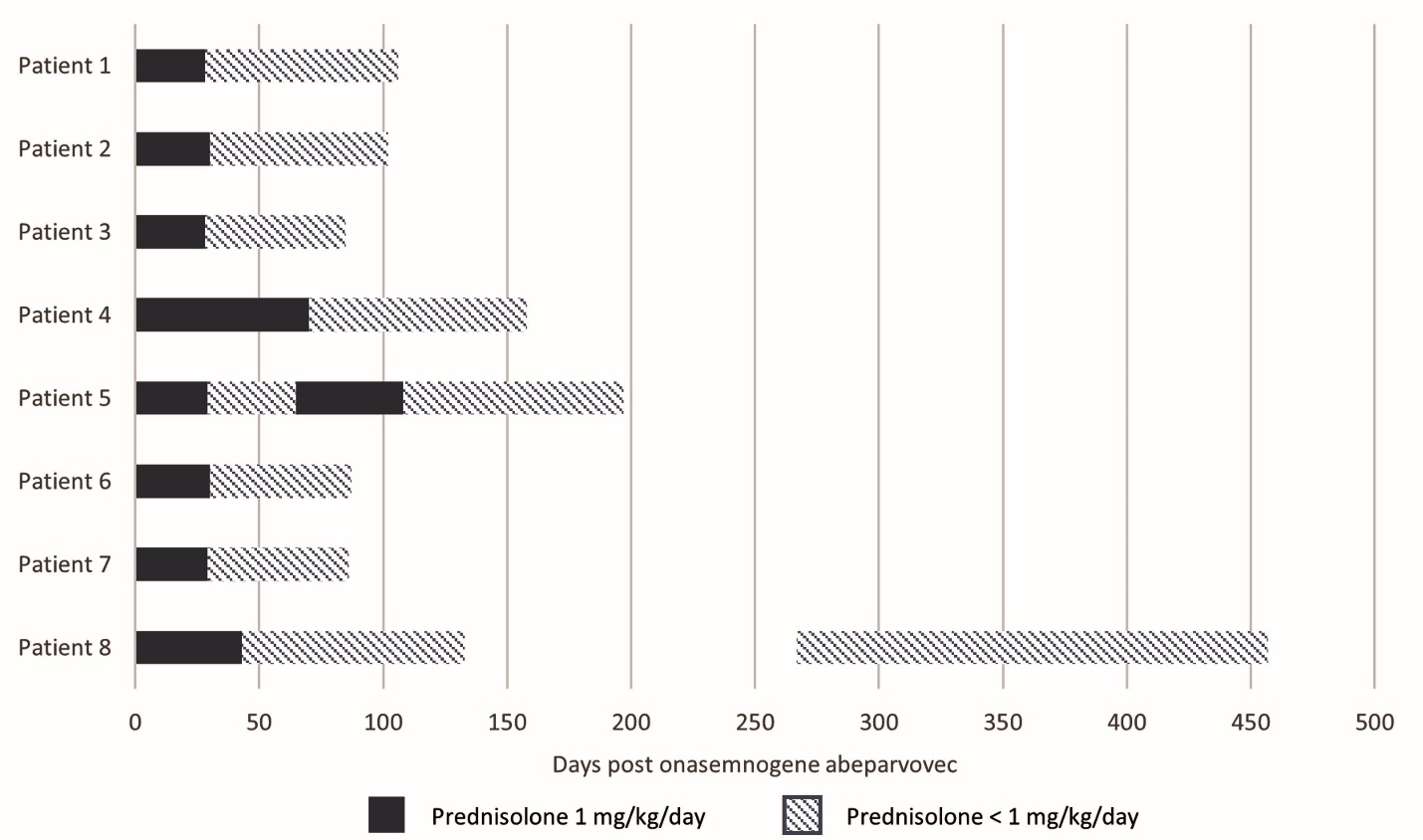

Supplement: Supplementary file 1 — supplementary material [file 41434_2025_535_MOESM1_ESM.docx]
